# Antimicrobial materials based on photothermal action and their application in wound treatment

**DOI:** 10.1093/burnst/tkae046

**Published:** 2024-12-04

**Authors:** Jiangli Cao, Zhiyong Song, Ting Du, Xinjun Du

**Affiliations:** State Key Laboratory of Food Nutrition and Safety, College of Food Science and Engineering, Tianjin University of Science and Technology, No.29, Thirteenth Street, Binhai New Area, Tianjin 300457, PR China; College of Sicence, Huazhong Agricultural University, No.1, Shizishan Street, Hongshan District, Wuhan 430070, PR China; State Key Laboratory of Food Nutrition and Safety, College of Food Science and Engineering, Tianjin University of Science and Technology, No.29, Thirteenth Street, Binhai New Area, Tianjin 300457, PR China; State Key Laboratory of Food Nutrition and Safety, College of Food Science and Engineering, Tianjin University of Science and Technology, No.29, Thirteenth Street, Binhai New Area, Tianjin 300457, PR China

**Keywords:** Multi-drug resistance bacteria, Nanoparticles, Anti-biofilm, Wound treatment, Photothermal therapy, Antimicrobial materials, Photothermal therapy

## Abstract

Considering the increasing abundance of antibiotic-resistant bacteria, novel antimicrobial approaches need to be investigated. Photothermal therapy (PTT), an innovative noninvasive therapeutic technique, has demonstrated significant potential in addressing drug-resistant bacteria and bacterial biofilms. However, when used in isolation, PTT requires higher-temperature conditions to effectively eradicate bacteria, thereby potentially harming healthy tissues and inducing new inflammation. This study aims to present a comprehensive review of nanomaterials with intrinsic antimicrobial properties, antimicrobial materials relying on photothermal action, and nanomaterials using drug delivery antimicrobial action, along with their applications in antimicrobials. Additionally, the synergistic mechanisms of these antimicrobial approaches are elucidated. The review provides a reference for developing multifunctional photothermal nanoplatforms for treating bacterially infected wounds.

## Graphical Abstract

**Graphical Abstract:**
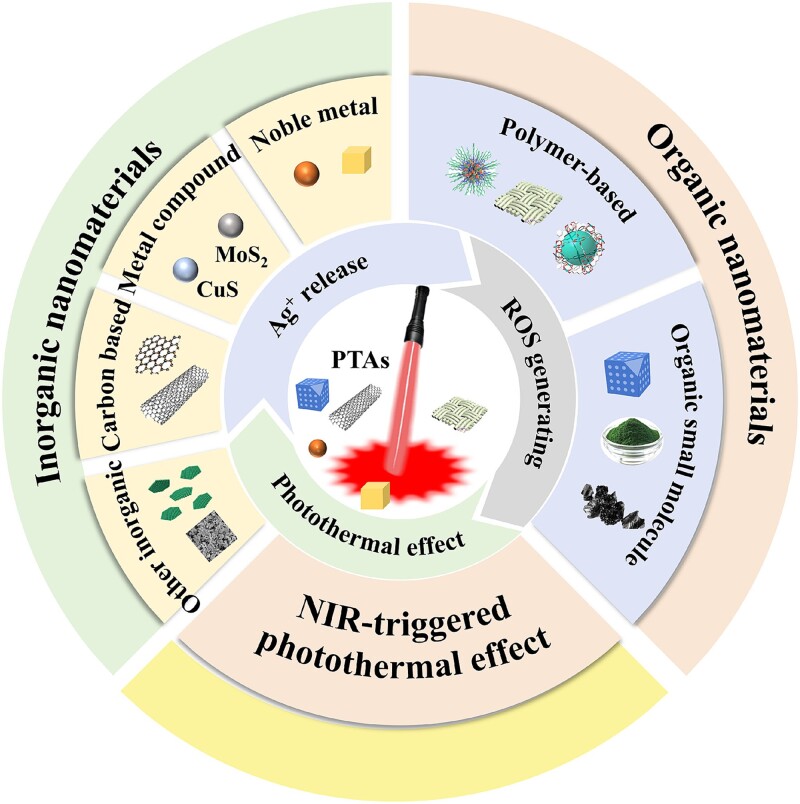

Highlights

- The focus is on the design and application of photothermal nanomaterials in antimicrobial and wound therapy.
- A comprehensive classification and discussion have been conducted on various types of inorganic and organic nanomaterials.
- The synergistic effects of different functionalized photothermal nanomaterials in antimicrobial and wound therapy are highlighted.

## Background

Bacterial infections pose a substantial public health threat, with bacterial biofilms being a major contributor to these infections. These biofilms significantly enhance bacterial resistance to antibiotics, exacerbating the issue. Hence, new strategies need to be developed to combat bacterial biofilms and improve the treatment of bacterial infections [1]. Also, the emergence of multidrug-resistant strains, often attributed to antibiotic misuse, has significantly contributed to the failure of antibiotic therapies and increased the demand for novel antimicrobial strategies. Recently, several antibiotic-independent physical therapies have shown increasing potential in the field of antimicrobials. These include antibacterial therapies based on bioactive substances [2,3], photodynamic therapy (PDT) [4], and photothermal therapy (PTT) [5]. Among these therapies, PTT has shown significant advantages in treating bacterial infections. The therapeutic principle of PTT involves photothermal agents (PTAs) that inhibit the development of drug-resistant bacteria and disrupt biofilm structures by converting light energy into heat energy. This conversion results in irreversible damage to bacterial cells, such as membrane rupture and protein denaturation [6]. Near-infrared (NIR) light irradiation, with a wavelength range of 700–1400 nm (and an optimal range of 700–950 nm), exhibits high photothermal conversion efficiency (PCE). In this range, biological tissues have low absorbance and light scattering, allowing deeper tissue penetration, lower absorption by water and hemoglobin, and minimal damage to normal tissues [7]. PTAs are crucial for the success of antibacterial PTT, making their selection and use vital. This study presents a comprehensive review of nanomaterials with intrinsic antimicrobial properties, antimicrobial materials relying on photothermal action, and nanomaterials using drug delivery mechanisms for antimicrobial action, along with their applications in antimicrobial therapies.

## Review

### Nanomaterials and antimicrobial applications

PTT is a promising therapeutic strategy for treating bacterial infections by rapidly generating large amounts of heat from a PTA under NIR light irradiation [8]. The main bacterial inhibition pathway of PTT is broadly divided into three steps as follows. (1) Targeting: PTAs are directed to the bacterial surface through various mechanisms, including electrostatic interactions, antibody recognition, and chemical group binding. (2) Heat generation: upon NIR light irradiation, PTAs convert absorbed light energy into thermal energy, generating localized heat on the bacterial surface. (3) Bacterial destruction: the bacterial surface proteins are denatured and the intracellular environment is disturbed by the increase in temperature, eventually leading to bacterial death [9]. Compared with conventional antibiotics, PTT has broad-spectrum antibacterial effects and short treatment duration, making it difficult for bacteria to develop resistance, thus achieving excellent antibacterial outcomes [10,11]. This study categorizes antimicrobial agents into materials with intrinsic antimicrobial properties, photothermal antimicrobial materials, and nanomaterials that rely on drug delivery for their antimicrobial effects, focusing on photothermal antimicrobial materials.

### Materials with intrinsic antimicrobial properties

#### Metallic antibacterial materials

Metallic antimicrobial materials have a wide range of applications and remarkable antimicrobial properties, and mainly include silver [12], copper [13], zinc [14], and gold [15]. Silver and silver nanoparticles (NPs) are widely used in medical devices, textiles and food packaging for their broad-spectrum antimicrobial properties [16–18]. Li prepared pectin/gelatin films loaded with curcumin and silver NPs as antimicrobial multifunctional food packaging films using a solution casting technique. The films showed 99.57 ± 0.16 and 100% inhibition of *Escherichia coli* and *Staphylococcus aureus*, respectively. As sustainable biomass-based materials, multifunctional composite films have a promising future in smart food-packaging applications [18]. Cu and its alloys act by penetrating bacterial cell walls with Cu ions, which alters the pH and function of membrane channels, disrupts cellular and inner membrane structures, and generates reactive oxygen species (ROS). This leads to irreversible damage such as protein oxidation, rupture of critical internal molecules such as enzymes, DNA, and RNA, and destruction of the entire cellular membrane through free-radical lipid peroxidation [19]. Zn and zinc oxide (ZnO) also produce ROS and are used in wound dressings and food packaging. Zn ions, after penetrating the bacterial cell membrane, rapidly hydrate to ZnO and produce ROS, which interact with the intracellular fluid and lead to cell death [20]. Li *et al*. reported that hydrogels loaded with ZnO NPs induced bacterial cell death by accumulating and disrupting DNA replication and ATP synthesis within the cell, affecting mitochondrial function, and destabilizing cell membranes, leading to membrane rupture and intracellular leakage [21]. Titanium dioxide has a photocatalytic effect under ultraviolet (UV) light and is used for self-cleaning surfaces and wound dressings. Mohammad *et al*. reported that adding essential oils to titanium dioxide provided strong antioxidant activity, maintaining cellular integrity and function at wound sites by reducing oxidative stress [22]. These metallic antimicrobial materials play an important role in medicine, industry, and everyday life.

Although metallic antimicrobial materials have demonstrated remarkable antimicrobial properties in a variety of fields, they present some drawbacks and challenges from a mechanistic perspective. First, Ag and Cu ions act as antimicrobials by disrupting bacterial cell membranes and metabolic processes; however, these ions may also be toxic to human cells, with prolonged exposure leading to health issues such as Ag toxicity (Ag deposition disorder) and Cu toxicity [23,24]. Additionally, nanoscale metal particles (e.g. Ag NPs and Cu NPs) may diffuse into the environment through water or soil, negatively impacting ecosystems and affecting microbial communities and biodiversity [25,26].

#### Polymer-based antibacterial materials

Polymer-based antimicrobial materials have shown remarkable results in wound healing. Among these, quaternary ammonium-containing polymers are cationic antimicrobial agents that can penetrate bacterial cells through electrostatic action, destroy electrolytes and disrupt cell membranes, thereby exerting a bactericidal effect. Hence, they are widely used in medical dressings [27]. Polymers doped with Ag NPs and Cu NPs are used in wound dressings and antimicrobial coatings and act by releasing metal ions that inhibit bacterial growth and disrupt cellular structures [28–31]. Additionally, natural polymers such as chitosan are essential in wound dressings due to their positive charge and interaction with bacterial cell walls, leading to cell membrane rupture [32,33]. Sodium alginate is also widely used to promote wound healing due to its biocompatibility and antimicrobial properties [34,35]. Other functionalized polymers, such as polyvinyl alcohol (PVA)–iodine complexes, exert an antimicrobial effect by oxidizing iodine and are suitable for antimicrobial wound dressings [36]. These polymer-based antimicrobial materials provide adequate antimicrobial protection through various mechanisms, promoting wound healing and reducing the risk of infection.

Although polymer-based antimicrobial materials have shown remarkable results in wound healing, they also present some drawbacks and challenges. First, the long-term use of quaternary-containing polymers such as poly(tetravinyl quaternary ammonium salt) and PVA-modified quaternary ammonium salt may lead to bacterial resistance issues [37]. Second, polymers doped with Ag NPs and Cu NPs, despite having excellent antimicrobial properties, may trigger cytotoxicity and environmental pollution. Therefore, the release of Ag and Cu ions needs to be strictly controlled to avoid potential harm to the human body and ecosystem. Natural polymers such as chitosan have good biocompatibility. However, they also need to be further optimized in terms of degradation rate *in vivo* and mechanical strength to ensure effectiveness and stability in the wound healing process [38]. Sodium alginate, though biocompatible, may dissolve in moist environments, affecting its physical stability and antimicrobial effect. Polymer-based antimicrobial materials have significant potential to promote wound healing. However, their potential toxicity, risk of drug resistance, environmental impact, and physical and chemical stability issues need to be carefully evaluated and addressed in practical applications.

#### Natural antibacterial materials

Chitosan is a common natural polysaccharide widely used as an antimicrobial nanomaterial in the medical field. It is extracted from the shells of crustaceans and has good biocompatibility and biodegradability [39]. It exhibits antimicrobial, antioxidant, and wound healing properties, making it a frequent component of medical dressings, drug delivery systems, and biomedical materials [40–43]. (1) Chitosan undergoes protonation under acidic conditions to form positively charged amino groups. It interacts with the negatively charged bacterial cell membrane through electrostatic interactions, disrupting the membrane. This disruption leads to osmotic imbalance, intracellular substance outflow, and ultimately, cell death, exerting an antibacterial effect. (2) Chitosan’s positive charge allows it to enter cells and interact with negatively charged proteins, inhibiting DNA replication mechanisms and disrupting bacterial physiological activities, achieving bactericidal and antibacterial effects. (3) Chitosan is rich in hydroxyl and amino functional groups. Also, it has excellent metal-ion adsorption ability under acidic conditions and can be used as a chelating agent. When inside the cell, chitosan surrounds metal complexes that impede the flow of essential nutrients such as calcium and Zn ions. These metal ions are vital for cell growth. Further, the chelating effect of chitosan inhibits the action of these metal ions, thus inhibiting cell growth and leading to cell death [44,45].

### Antibacterial materials relying on the photothermal effect

#### Noble metal nanomaterials

Precious metals, such as Au [46], Ag [47], platinum [48], ruthenium (Ru) [49], and palladium [50,51], are often considered as inert materials. However, these metallic nanomaterials exhibit significant localized surface plasmon resonance (LSPR) properties. This means they absorb light energy at specific wavelengths, generating oscillating free electrons at the surface, thereby generating heat to induce bacterial death. Consequently, they are attractive candidates for antibacterial nanomaterials [52]. Additionally, different sizes, morphologies, and interparticle coupling lead to different heat-production capabilities of metal nanomaterials. The LSPR band can be optimized by adjusting the aforementioned factors to achieve thermal relaxation under appropriate irradiation and generate high temperatures [53]. The noble metal-based photothermal nanomaterials and their comparative antibacterial activities are summarized in Table 1.

**Table 1:** Comparison of antibacterial activity of noble metal-based photothermal nanomaterials

Nano-complexes	Size	Antibacterial mechanism	NIR laser	Bacteria	Effect	Ref.
GNR@LDH-PEG	200 nm	PTT	808 nm 2.0 W/cm^2^	*E. coli* *S. aureus*	99.25% 88.44%	[54]
NiFe_2_O_4_@Au/PDA	250 nm	PTT/magnetolytic force	808 nm 2.0 W/cm^2^	*E. coli* *S. aureus*	~100%	[55]
AuNR@SiO_2_@UiO-66	~200 nm	PTT	NIR Light 224 mW/cm^2^	*E. coli* *S. aureus*	–	[56]
FePAgPG	20 nm	PTT/Ag^+^/magnetolytic force	808 nm 0.75 W/cm^2^	*S. mutants*	>95%	[57]
Ag_2_O_2_ NPs	43 ± 10 nm	PTT/Ag^+^/US	808 nm 0.7 W/cm^2^	MRSA *S. aureus* *P. aeruginosa* *E. coli*	99.9999%	[47]
KCT@PCN-Ag	–	PTT/PDT/Ag^+^	780 nm 31.45 W/cm^2^	*E. coli* *S. aureus*	99.9999% 99.928%	[58]
dvPtNPs	1.5 nm	PTT/PDT/CDT	808 nm 1.5 W/cm^2^	*E. coli* MRSA	–	[48]
Au@Pt NRs	5 nm	PTT	808 nm 3 W/cm^2^	*S. aureus* *P. aeruginosa*	100% 87.2%	[59]
AA@Ru@HA-MoS_2_	~100 nm	CDT/PTT	808 nm 0.5 W/cm^2^	*MDR S. aureus* *MDR P. aeruginosa*	89.2% 81.9%	[49]
[Ru(CO)_2_Cl_2_]*_n_*		PTT	365 nm 1.0 kW-h/m^2^	*S. aureus* *E. coli*	100% 99.999%	[60]
MXene@AgPd/PDA		PTT/nanozymes	808 nm 2.5 W/cm^2^	*S. aureus* *E. coli*		[61]
PdH	20 nm	H_2_/PTT	808 nm 1.5 W/cm^2^	*S. aureus* *E. coli*	~100%	[62]
Pd-Cu/AMO@ZIF-8	196.3 nm	PTT/CDT	808 nm 1.0 W/cm^2^	*S. aureus* *P. aeruginosa*	99.8% 99.1%	[63]

**Figure 1:**
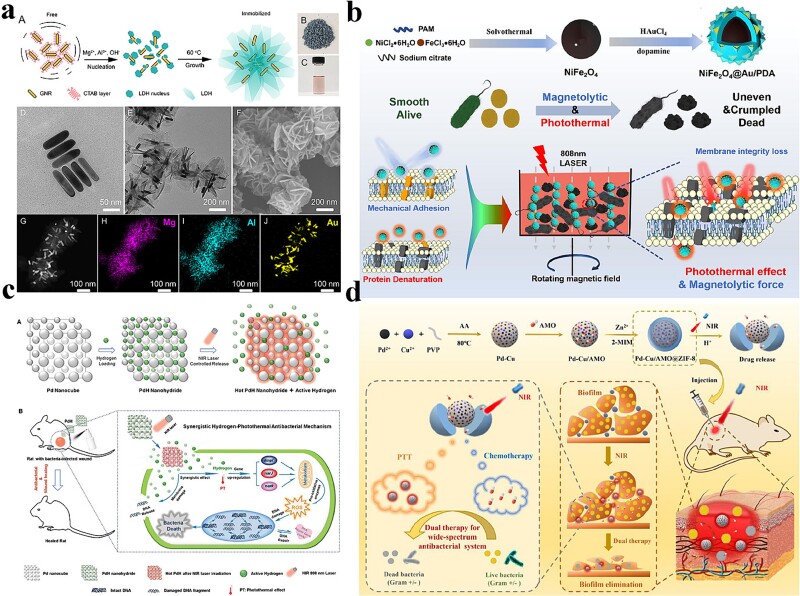
Antibacterial activity of noble metal-based photothermal nanomaterials. (**a**) Preparation and characterization of GNR@LDH. Adapted with permission [54]. Copyright © 2019, American Chemical Society. (**b**) Antibacterial mechanism of NiFe_2_O_4_@Au/PDA. Adapted with permission [55]. Copyright © 2022 Elsevier B.V. All rights reserved. (**c**) Antibacterial mechanism of PdH. Adapted with permission [62]. Copyright © 1999–2023 John Wiley & Sons, Inc. All rights reserved. (**d**) Preparation and *in vivo*/ex *vivo* antibacterial mechanism of Pd-Cu/AMO@ZIF-8. Adapted with permission [63]. Copyright © 2021 Acta Materialia Inc. Published by Elsevier Ltd. All rights reserved. *PDA* polydopamine

Different configurations of Au-based nanomaterials have gained interest with the increasing use of antimicrobial PTT. Among these, Au nanorods (GNR) have been widely studied for their advantages, such as a wide aspect ratio and high absorbance. GNRs exhibit two types of plasmonic excitations: a transverse peak at 520 nm and a vertical peak located in the NIR domain [52]. Ma *et al*. adjusted the photothermal conversion efficiency of GNRs by increasing the length-to-diameter ratio and forming a core–shell structure. Also, GNRs were combined with layered double-hydroxide (LDH) to construct the core–shell GNR@LDH nanostructure (Fig. 1a). The interaction between Au and LDH resulted in a certain number of electron defects on the Au surface, leading to increased heat energy conversion. Under 808-nm laser irradiation, the GNR@LDH exhibited a remarkable η value of 60%, indicating significantly improved photothermal conversion efficiency compared with other single GNRs. Moreover, the use of cetyltrimethylammonium bromide (CTAB) was circumvented in the synthesis process, mitigating the cytotoxicity of the nanomaterials [54].

Other researchers were not satisfied with employing a single form of PTT for antimicrobial purposes, but instead utilized multiple modes of antimicrobial activity, thus enhancing the efficiency and specificity of noble metal nanomaterials in combating bacteria. Common strategies included combining metal compound nanomaterials, antibiotics, bacteria-trapping agents, or photodynamic agents with noble metal-based nanomaterials. These combined approaches provided multiple mechanisms for eradicating target bacteria [64–66]. Xu *et al*. investigated a NiFe_2_O_4_@Au/PDA hybrid material obtained by covering Au and polydopamine (PDA) layers on stable magnetic NiFe_2_O_4_ nanospheres (Fig. 1b). This hybrid material exhibited excellent magnetic resolution toward biological organisms under a rotating external magnetic field. Furthermore, its photothermal antibacterial performance was significantly enhanced when exposed to a rotating magnetic field [55]. Magnetic enrichment and photothermal co-action improved the therapeutic effect, allowing for precise localization to the site of infection and minimizing damage to the surrounding tissues. Meanwhile, the presence of magnetic nanomaterials enhanced the photothermal effect, making the treatment more durable and potent. However, the long-term safety and biocompatibility of magnetic nanomaterials require further research and validation to ensure the safety and reliability of the treatment process. To address safety and reliability issues, Han *et al*. developed a composite material using a metal–organic framework (MOF), in which the outer shell GNR produced a photothermal effect when irradiated with NIR light, whereas the internal iodine was actively released due to induced heat. This approach provided a promising strategy for the triggered release of iodine and offered potential benefits in antimicrobial applications [56]. Besides the aforementioned nanomaterials, some forms such as Au nanocages, Au nanoflowers, Au nanostars, and AuNPs were also used for antimicrobial PTT [67–70].

Ag nanomaterials have been extensively studied as antimicrobial agents. Xu *et al*. used a PDA layer in mitigating the excessive release of Ag^+^, thereby reducing damage to normal tissues. FePAgPG NPs showed excellent pH-responsive targeting of pathogens in the acidic microenvironment, removing >95% of cariogenic bacteria at 53°C for 10 min. Finally, these nanomaterials were conveniently removed using magnetic fields [57]. Similarly, Bi *et al*. synthesized Ag_2_O_2_ NPs that enabled the controlled release of ROS, thereby mitigating the toxic effects of nanomaterials on healthy tissues. This innovative approach allowed for regulated ROS release, ensuring a safer and more targeted application of nanomaterials in biological systems. Ag^+^ and ROS were produced by exogenous ultrasound- and NIR light-regulated stimuli. This study showed that Ag_2_O_2_ NPs had strong antibacterial properties and biofilm-formation abilities, killing >99.9999% of bacteria within 10 min and significantly promoting the healing of methicillin-resistant *S*. *aureus* (MRSA)-infected skin wounds [47]. Nie *et al*. developed a self-sterilizing textile with synergistic PDT and PTT properties to combat microbial infections in hospitals and medical institutions. This textile allowed for a continuous release of Ag ions. The team successfully grew porphyrin metal–organic backbone (PCN-224) and Ag NPs *in situ* on knitted cotton textiles. This resulted in rapid photodynamic antibacterial effects and long-lasting bacterial inhibition. The monomorphic oxygen (^1^O_2_) produced by PCN-224 under light promoted further degradation of Ag NPs and released more Ag^+^ for antibacterial action, achieving long-lasting bacterial inhibition [58].

Considering the photothermal antibacterial efficiency of Au- and Ag-based nanomaterials, researchers also evaluated the photothermal antibacterial ability of Pt. Deng *et al*.’s design involved novel bivalent Pt NPs (dvPtNPs) composed of Pt^0^ cores and Pt^2+^ shells. The Pt^0^ cores of dvPtNPs were capable of efficient photothermal conversion, enabling effective thermal therapy when exposed to NIR light. Simultaneously, the Pt^2+^ shells released ROS and chemotherapeutic Pt^2+^ ions, adding photodynamic and chemotherapeutic effects to the antimicrobial action. This multifunctional approach combined three modes of antimicrobial action: photothermal (thermal therapy), photodynamic (ROS-mediated damage), and chemotherapeutic (Pt^2+^ ions) [48]. Based on the excellent photothermal effect and antibacterial ability of GNRs, Zhang *et al*. prepared a highly efficient photothermal agent Au@Pt NRs by growing Pt nanodots on the surface of GNRs. The LSPR absorption band of Au@Pt NRs could be adjusted in the range 755–845 nm compared with that of GNRs. Under NIR irradiation, Au@Pt_0.1_ NRs exhibited extremely high photothermal lysis efficiency for bacteria. Additionally, the introduction of Pt nanodots enhanced the affinity of nanomaterials for bacteria and significantly reduced cytotoxicity [59].

Ghosh *et al*.’s research focused on addressing the limitations of ruthenium ligand compounds, which possess promising anticancer and antibacterial activities but suffer from poor water solubility and low stability under physiological conditions. In their study, Ghosh *et al*. designed a polymeric precursor called dicarbonyl dichlororuthenium(II) {[Ru(CO)_2_Cl_2_]*_n_*} to investigate its antibacterial activity. The unique aspect of this study is that the carbonyl ruthenium precursor, [Ru(CO)_2_Cl_2_]*_n_*, is a polymeric compound without any organic ligands. The use of this precursor eliminates the need for organic ligands, which are often a contributing factor to the poor water solubility and low stability of traditional ruthenium compounds. Importantly, [Ru(CO)_2_Cl_2_]*_n_* polymers inhibit bacterial growth by disrupting bacterial membranes and up-regulating the stress response [60]. Transition-metal disulfide (TMD)-based nanomaterials have been widely explored for PTT. However, existing TMD nanomaterials do not have significant absorption peaks in the NIR region, limiting their efficacy for application [71]. To address this issue, Liu *et al*. used mesoporous Ru NPs as nanocarriers loaded with the pre-drug ascorbic acid (AA) and encapsulated with hyaluronic acid (HA). Molybdenum disulfide (MoS_2_) precoated with ciprofloxacin was then used as a targeting catalyst to bind to HA to form the AA@Ru@HA-MoS_2_ nanosystem. When this nanosystem accumulated at the site of infection, the bacterially secreted hyaluronidase degraded HA and triggered the release of AA, which then generated hydroxyl radicals (·OH) *in situ* catalyzed by MoS_2_. In addition, the good photothermal properties of Ru NPs were used to achieve combined chemodynamic therapy (CDT) and PTT antibacterial activity [49].

Nanoscale Pd materials also exhibited strong LSPR adsorption in the NIR region, providing excellent photothermal conversion properties for PTT [72,73]. Jin *et al*. designed AgPd bimetallic nanocrystals with cage-like nanostructures sandwiched between transition metal carbide/nitride (MXene) nanosheets (NSs) and PDA layers to achieve high catalytic efficiency and antibacterial properties. Under NIR irradiation (808 nm and 2.5 W/cm^2^), the catalytic kinetics of MXene@AgPd/PDA nanomaterials improved ~1.2-fold [61]. In addition, their antibacterial activity was further enhanced under NIR light irradiation due to the NIR photothermal effect. Some scholars achieved synergistic antibacterial effects by combining noble metal-based nanomaterials with gases such as NO and H_2_ [74–76]. Yu *et al*. synthesized a biocompatible hydrogen-releasing nanohydride, PdH, by doping H_2_ into Pd nanocubes. This nanohydride demonstrated controlled and on-demand active hydrogen release properties when exposed to NIR light (Fig. 1c). The resulting PdH nanohydride combined the benefits of bioactive hydrogen with the photothermal effect of Pd, showcasing outstanding antimicrobial activity both *in vitro* and *in vivo*. This study presented a promising avenue for leveraging the synergistic effects of hydrogen and PTT for antimicrobial applications. [62]. Furthermore, in-depth antimicrobial mechanistic studies suggested that the synergistic hydrogen-photothermal antimicrobial effect involved two potential mechanisms. The first mechanism involved the upregulation of bacterial metabolism-related genes, such as *dmpI*, *narJ*, and *narK*. This upregulation led to increased expression of oxidative metabolic enzymes, resulting in higher production of ROS. The elevated levels of ROS induced DNA damage within the bacterial cells. The second mechanism involved the induction of severe damage to the bacterial cell membrane, leading to the release of intracellular compounds, including DNA, from the bacteria.

Wang *et al*. developed a dual-stimulus-responsive activated procedural antimicrobial system that responded to NIR light and pH changes. The system involved synthesizing and encapsulating a Pd–Cu nanoalloy (PC) and the antibiotic amoxicillin (AMO) within zeolitic imidazolate framework-8 (ZIF-8). The system released AMO in an acidic environment, disrupting the bacterial cell wall. The rate of AMO release decreased over time. The temperature increased on NIR irradiation, triggering further drug release and completing the antimicrobial action [63]. Overall, this system offered a promising strategy for developing effective and safe treatments for bacterial infections. The combined effects of pH and NIR stimuli provided a comprehensive approach to combating bacterial infections.

#### Metal compound nanocomposites

Precious metal nanomaterials present significant challenges for biomedical applications because of their high cost and potential long-term cytotoxicity. In contrast, metal sulfides and oxides have gradually gained the attention of researchers because of their good biocompatibility, low manufacturing cost, and good photothermal conversion efficiency. Table 2 provides a comparison of various metal compound photothermal nanomaterials and their antibacterial activities.

**Table 2:** Comparison of antibacterial activity of metal compound photothermal nanomaterials

Nano-complexes	Size	Antibacterial mechanism	NIR laser	Bacteria	Effect	Ref.
CS@MoS_2_-Ti	60 nm	PTT&PDT	880 nm 660 nm	*E. coli* *S. aureus*	99.84% 99.65%	[77]
MoS_2_/PDA/RGD	7–10 nm	PTT	- 0.5 W/cm^2^	*E. coli* *S. aureus*	99.2%	[78]
CuS NDs	4 nm	PTT	808 nm 2.0 W/cm^2^	*E. coli* MRSA	–	[79]
CuS/mSiO_2_-MPS NPs	–	PTT&PDT	808 nm 2.0 W/cm^2^	*E. coli* *S. aureus*	95.91% 88.87%	[80]
Bi_2_S_3_: Gd@Cu-BIF	163 nm	PTT/CDT	808 nm 1.0 W/cm^2^	*E. coli* MRSA	99.99%	[81]
BSA-BiZ/CuxS NCs	10 ~ 20 nm	PTT/PDT	808 nm 0.53 W/cm^2^	*Salmonella enteritidis* *MRSA* *S. aureus* *P. aeruginosa* *E. coli*	> 90%	[82]
CuS@p-DMSNs	156 nm	PTT	1064 nm 1.5 W/cm^2^	*E. coli* *S. aureus*	~100%	[83]
Ag/CS@ MnO_2_-Ti	–	PTT	808 nm 0.5 W/cm^2^	*E. coli* *S. aureus*	99.25% 99.00%	[84]
ND	112 nm	PTT/nanozymes	808 nm 0.75 W/cm^2^	*E. coli* MRSA	100%	[85]
PDA@FeS NPs	97.3 nm	PTT/PDT	808 nm 1.0 W/cm^2^	*E. coli* *S. aureus*	100% 99.5%	[86]
CoFe-LDH	50 nm	PTT/CDT	808 nm	*E. coli* *S. aureus*	~100%	[87]
CeO_2_/Nb_2_C		PTT/nanozymes	808 nm 1.0 W/cm^2^	*E. coli* *S. aureus*	93% 98%	[88]

Molybdenum disulfide (MoS_2_) is widely explored in TMDs due to its large surface area, high biocompatibility, high NIR absorption, and low cytotoxicity [89]. MoS_2_-based nanomaterials have demonstrated the ability to effectively suppress bacterial growth through multiple mechanisms, including contact killing, PDT, and PTT. Feng *et al*. activated the photothermal and photodynamic effects of chitosan-assisted MoS_2_ (CS@MoS_2_) within a short timeframe using a dual-wavelength laser at 660/808 nm [77]. Besides the good photothermal effect of MoS_2_, its inherent ROS-independent oxidative stress damage was even more effective in bacterial inhibition. Yuan *et al*. prepared functional MoS_2_/PDA-arginine-glycine-aspartic acid (RGD) coatings on Ti implants, which enabled the Ti substrate to have effective antibacterial ability under NIR irradiation. These coatings also accelerated glutathione oxidation by photothermal heating, inducing oxidative stress derived from ROS inherent in MoS_2_ NSs; this disrupted bacterial membrane integrity, synergistically causing protein leakage and ATP reduction [78].

**Figure 2:**
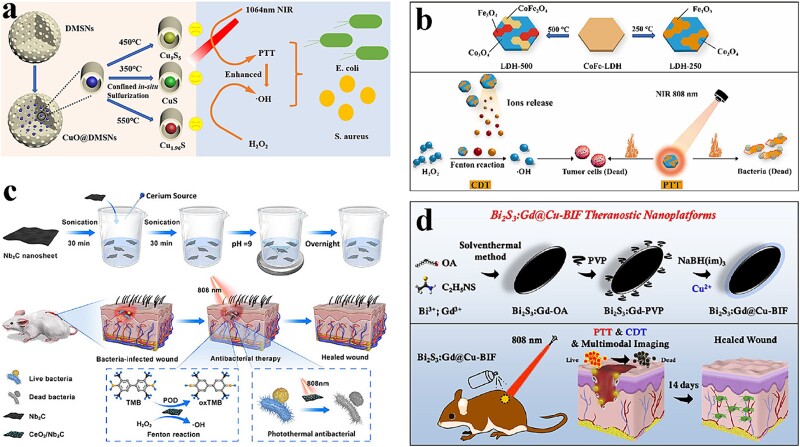
Antibacterial activity of metal compound nanocomposites photothermal nanomaterials. (a) Preparation and characterization of CuS@p-DMSNs. Adapted with permission [83]. Copyright © 2023 Elsevier B.V. All rights reserved. (b) Antibacterial mechanism of CoFe-LDH. Adapted with permission [87]. Copyright © 2023 Elsevier B.V. All rights reserved. (c) Schematic diagram of the CeO_2_/Nb_2_C nanocomposite used to kill pathogens. Adapted with permission [88]. Copyright © 2023, American Chemical Society. (d) Antibacterial mechanism of Bi_2_S_3_:Gd@Cu-BIF. Adapted with permission [81]. Copyright © 2022 Acta Materialia Inc. Published by Elsevier Ltd. All rights reserved. *PTT* photothermal therapy

Copper sulfide (CuS) is commonly used as a metal sulfide for antimicrobial purposes. It is often combined with biocompatible polymers such as bovine serum albumin (BSA) to generate nanocomposites. Qiao *et al*. developed ultra-small CuS nanodots (CuS NDs) as part of a bifunctional nanosystem designed to treat chronic nonhealing wounds infected with multidrug-resistant bacteria [79]. This nanosystem demonstrated the ability to effectively eradicate multidrug-resistant bacteria and accelerate wound recovery by harnessing the photothermal effect and remotely controlling the release of Cu^2+^. The released Cu^2+^ played a key role in promoting fibroblast migration and vasculogenesis of endothelial cells, thereby accelerating the wound healing process. Li *et al*. synthesized mesoporous silica-modified CuS NP heterogeneous hydrogels via a free-radical polymerization process. This material exhibited remarkable photothermal and photodynamic properties, which could be precisely controlled on 808-nm NIR light irradiation. The antibacterial effects against *S. aureus* and *E. coli* were measured at 99.80 and 99.94% reduction in bacterial growth, respectively, within just 10 min [80]. These findings underscored the potential of CuS-based nanosystems and hydrogels in combating multidrug-resistant bacteria, facilitating wound healing, and achieving highly effective antibacterial effects through modulating photothermal and photodynamic properties. Bi_2_S_3_ is widely used because of its remarkable properties, such as good biodegradability, easy composition, and exceptional photothermal convertibility. Qi *et al*. developed a novel nanocomponent, Bi_2_S_3_:Gd@Cu-boron imidazolate framework (BIF), targeting the microenvironment of bacterial infections, that exhibited significant antibacterial efficacy against *E. coli* and MRSA both *in vitro* and *in vivo* (Fig. 2d). This nanocomplex demonstrated a synergistic effect of photothermal and chemical kinetics (PTT/CDT) in accelerating wound healing post-MRSA infection [81]. Nain *et al*. synthesized a bismuth–CuS nanocomposite, BSA-BiZ/CuxS NCs, using BSA as a template. These NPs exhibited broad-spectrum antibacterial activity against multidrug-resistant bacterial strains and biofilms upon NIR irradiation due to their favorable catalytic, photodynamic, and photothermal properties [82]. Xu *et al*. proposed a confined sulfidation strategy to fabricate CuS-loaded dual mesoporous silica nanospheres (CuxSy@DMSNs) with various crystalline phases, enabling reactive ROS-mediated and photothermal antimicrobial applications (Fig. 2a). The controlled crystalline phases of CuS and adjustable sulfidation temperature provided tailored antimicrobial properties while maintaining minimal cytotoxicity to normal cells *in vitro* [83]. These studies demonstrate promising antibacterial efficacy and potential for clinical applications. However, further investigations are needed to assess the long-term safety, biocompatibility, and scalability of these nanocomponents. Additionally, more comprehensive studies involving diverse bacterial strains and infection models are necessary to validate their broad-spectrum antibacterial activity and therapeutic efficacy. Moreover, comparative studies with existing antibacterial agents might provide insights into the superiority of these nanocomponents and their potential clinical utility.

Other metal oxides such as MnO_2_ can also be used for antibacterial PTT. Wang *et al*. found that exogenous NIR irradiation stimulated MnO_2_ NSs to generate sufficiently high temperatures in just 10 min. This, combined with the small level of pre-released Ag^+^ in the coating, contributed to excellent antimicrobial efficiency through a synergistic effect [84]. Wang *et al*. constructed a biodegradable nickel disulfide (ND) nanoenzyme, which effectively catalyzed the decomposition of H_2_O_2_ to produce ·OH via photothermal effect. The high temperature of the ND nanoenzyme produced using PTT further enhanced its catalytic activity with no significant toxicity *in vivo* [85]. Ferrous and sulfur ions play a vital role in maintaining normal human physiology. Xu *et al*. employed a straightforward pre-doped polymerization–co-precipitation strategy to design and synthesize PDA NPs embedded with ferrous sulfide (PDA@FeS NPs). The smart PDA array effectively protected FeS NPs from oxidation and aggregation. Incorporating PDA and ferrous sulfide resulted in a significant synergistic enhancement of the photothermal effect. Hence, the PDA@FeS NPs exhibited significant photothermal bacterial resistance against both *E. coli* and *S. aureus* [86]. CDT has been extensively investigated for antitumor applications. Xu *et al*. prepared CoFe-LDHs by a co-precipitation method based on the synergistic effect of PTT and PDT, imparting photothermal and chemodynamic properties to CoFe-LDH (Fig. 2b). Bacteria cultured with LDH-250 and LDH-500 were completely killed under NIR irradiation [87]. Yuan *et al*. designed a CeO_2_/niobium carbide (Nb_2_C) nanocomposite with dual functions, including peroxidase activity and excellent NIR thermal properties. This nanocomposite was specifically tailored for applications in diabetic wounds (Fig. 2c). Under 808-nm laser irradiation, the CeO_2_/Nb_2_C nanocomposite exerted photothermal antimicrobial effects and exhibited synergistic catalytic properties, resulting in sustained bactericidal activity of >80% [88].

One potential drawback of using metal-oxide-based antibacterial PTT is the risk of cytotoxicity and tissue damage associated with high temperatures generated during the treatment process. Although the aim is to selectively target and eliminate bacteria, excessive heating may also cause damage to surrounding healthy tissues. Additionally, the long-term biocompatibility and potential systemic effects of metal-oxide NPs need a thorough investigation to ensure their safety for clinical applications. Moreover, challenges may exist related to the scalability and cost-effectiveness of manufacturing metal-oxide-based PTT agents for widespread clinical use. Therefore, comprehensive studies addressing these concerns are essential to advance the translation of metal-oxide-based PTT from bench to bedside.

#### Carbon-based nanomaterials

Carbon-based nanomaterials, including graphene-based nanomaterials (GBNs), carbon nanotubes (CNTs), and carbon dots, have garnered significant interest in the research community. These materials are extensively investigated due to their remarkable bactericidal efficacy combined with low cytotoxicity. Their distinctive characteristics, such as high specific surface area, excellent thermal and electric conductivity, and exceptional optical properties, make them particularly suitable for PTT applications [90,91]. Table 3 summarizes the comparison of carbon-based photothermal nanomaterials and their antibacterial activities.

**Table 3:** Comparison of the antibacterial activity of carbon-based photothermal nanomaterials

Nano-complexes	Size	Antibacterial mechanism	NIR laser	Bacteria	Effect	Ref.
GCS-CG	–	PTT/bacteria capture	808 nm 0.75 W/cm^2^	*E. coli* *S. aureus*	~100%	[92]
rGO/AuNS	78.3 nm	PTT	808 nm 3.0 W/cm^2^	*E. coli* *S. aureus*	100%	[93]
Au-QCMC-GO	–	PTT/bacteria capture	808 nm 4.0 W/cm^2^	*E. coli* *P. aeruginosa* *B. subtilis* *S. aureus*	–	[94]
rGB/QCS/PDA-PAM	–	PTT/bacteria capture	808 nm 0.8 W/cm^2^	MRSA *E. coli*	94.6% 96.6%	[95]
B-CG-QAS	223 nm	PTT/bacteria capture	808 nm 0.5 W/cm^2^	*multidrug resistance (MDR) Acinetobacter baumannii* *MDR P. aeruginosa* *MDR K. pneumonia*	>95%	[96]
N-GQD	5 nm	PTT	808 nm 0.4 W/cm^2^ 1064 nm 1.0 W/cm^2^	MRSA *E. coli* *S. aureus*	>97%	[97]
Fe_3_O_4_-CNTs-PNIPAM	–	PTT/bacteria capture/magnetic recycle	808 nm 3.0 W/cm^2^	*E. coli* *S. aureus*	~100%	[98]
PF127/CNT	–	PTT	808 nm -	*E. coli* *S. aureus*	~100%	[99]
CS/CNT/HA	–	PTT	808 nm 1.0 W/cm^2^	*E. coli* *S. aureus*	~75%	[100]

More typical nanomaterials such as GBNs are widely explored. GBNs have sharp edges that can disrupt the bacterial cell membranes, leading to the leakage of bacterial contents and achieving a bactericidal effect [101]. A single type of nanomaterial can no longer meet the bactericidal requirements and hence needs to be assembled with other functional molecules or nanostructures for better bactericidal activity. Qian *et al*. designed a novel nanomaterial called glycol chitosan-conjugated carboxygraphene (GCS-CG) by combining functional molecules and nanostructures to enhance bactericidal efficiency. GCS-CG was surface-adapted and biocompatible, demonstrating pH-responsive behavior with a rapid shift in surface charge from negative to positive. It showed self-adaptation to the acidic microenvironment of an abscess under acidic conditions, while causing no harm to the healthy tissue surrounding the abscess under neutral conditions. Furthermore, the conjugation of CG with GCS resulted in a significant increase in absorbance in the NIR region, leading to enhanced heat generation by GCS-CG [92]. Thus, it exhibited a strong antibacterial effect. Feng *et al*. used a seed-mediated growth method to prepare 2D reduced graphene oxide-loaded Au nanostar composites (rGO/AuNS). These composites exhibited good antibacterial activity due to the spines and sharp edges of the nanostructures, with a low cell viability of 32% when incubated for 180 min under light-free conditions. The bactericidal efficiency significantly increased under 808-nm NIR laser irradiation due to the local high-temperature effect of rGO/AuNS, thus achieving 100% bacterial inhibition [93]. Luo *et al*. investigated a functionalized paper for NIR laser-triggered photothermal ablation of infectious pathogens, which was formed by conjugating Au NPs and graphene oxide. Under NIR laser irradiation, the Au-quaternized carboxymethyl chitosan (QCMC)-GO (+)/nano-cellulose paper warmed up to >80°C, which was sufficient to inhibit the growth of gram-positive bacteria (*Bacillus subtilis* and *S. aureus*) and gram-negative bacteria (*E. coli* and *Pseudomonas aeruginosa*); the paper product could be used for skin or medical device disinfection [94].

**Figure 3:**
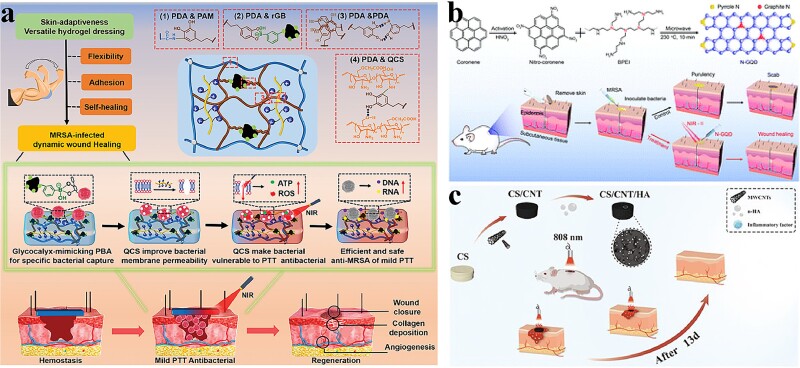
Antibacterial activity of carbon-based nanocomposites photothermal nanomaterials. (**a**) Schematic diagram of rGB/QCS/PDA-PAM hydrogel for accelerating wound healing in methicillin-resistant *Staphylococcus aureus* infection at low temperature (< 50°C). Adapted with permission [95]. Copyright © 1999–2023 John Wiley & Sons, Inc. All rights reserved. (**b**) Antibacterial mechanism of N-GQD. Adapted with permission [97]. Copyright © Royal Society of Chemistry 2023. (**c**) Schematic diagram of the CS/CNT/HA used to kill pathogens. Adapted with permission [100]. Copyright © 2023 Oxford University Press and Chinese Society for Biomaterials. *GQD* graphene quantum dots, *PDA* polydopamine

Zhao *et al*. achieved dynamic wound adaptation and mild photothermal antimicrobial activity based on a previous study combining functionalized graphene with hydrogels (Fig. 3a). Specifically, the surface of phenylboronic acid-functionalized graphene (rGB) mimicked phenylboronic acid to resemble the glycocalyx, enabling the hydrogel to selectively trap bacteria in a specific manner. This approach held significant promise in the field of wound treatment and infection control [95]. Wang *et al*. developed a dual-targeted antibacterial platform called B-CG-QAS based on boronic acid-functionalized graphene-based quaternary ammonium salts. This platform exhibited synergistic chemotherapeutic–photothermal effects. When positioned at the site of bacterial infection, B-CG-QAS selectively bound to the bacterial surface and its biofilm through the combined actions of electrostatic adhesion and covalent coupling. It enhanced bactericidal efficacy and had a stronger targeting ability than single-target drugs (B-CG or CG-QAS) [96].

Graphene quantum dots (GQDs) are considered promising photothermal inorganic nanomaterials due to their unique properties. One significant advantage of GQDs is their ability to be easily eliminated from the body, making them suitable for biomedical applications. Additionally, GQDs exhibit low biotoxicity, making them safer for use in biological systems. Furthermore, they possess inherent antibacterial properties, particularly in the blue light region with a wavelength range of 400–470 nm. This characteristic expands their potential in antibacterial applications, as they can be used for targeted bacterial eradication using specific wavelengths of light. Geng *et al*. synthesized highly graphitic-N-doped GQDs (N-GQDs) with efficient NIR-II photothermal conversion properties for photothermal antimicrobial therapy (Fig. 3b) [97].

CNTs, another type of carbon-based nanomaterial, also possess photothermal conversion capabilities. Yang *et al*. controlled the capture, ablation, and release of pathogenic bacteria through a stimulated response modulation mechanism. When the temperature increased to >15°C, poly (N-isopropylacrylamide) (PNIPAM) shifted from hydrophilic to hydrophobic. Bacteria can be effectively immobilized in it and then killed using PTT. After cooling, a magnetic field was used to recover the nanomaterials, thus removing the trapped and dead bacteria [98]. He *et al*. designed a nanocomposite hydrogel with unique attributes, including conductivity, self-healing ability, adhesion, and notable photothermal antimicrobial properties. The hydrogel was developed using *N*-carboxyethyl chitosan combined with benzaldehyde-capped Pluronic F127/CNTs (PF127/CNT). This combination resulted in a versatile hydrogel with enhanced conductivity, the ability to self-repair, strong adhesion, and significant photothermal antimicrobial activity [99]. Wang *et al*. focused on the development of chitosan (CS)-based porous scaffolds with enhanced wound healing properties. Three different scaffold types were created by the freeze-drying method: CS, CS/CNT (carbon nanotubes), and CS/CNT/HA (nanohydroxyapatite) (Fig. 3c). The addition of CNTs to the scaffolds resulted in improved mechanical properties, excellent photothermal response, and increased *in vitro*/*in vivo* photothermal antimicrobial activity. The CS/CNT scaffold exhibited excellent healing properties as a wound dressing in a mouse all-skin wound model, leading to enhanced wound closure, collagen deposition, and angiogenesis. Among these, the CS/CNT/HA combination scaffold exhibited a significant capacity to foster whole-skin wound closure and skin renewal, indicating its potential for effective wound healing applications [100].

A potential challenge associated with the use of CNTs for photothermal antimicrobial therapy is their propensity for aggregation and poor dispersion in physiological environments. CNTs may aggregate and settle out of solution due to their high aspect ratio and tendency to form bundles, leading to uneven distribution and reduced efficacy of the photothermal treatment. This aggregation could hinder their ability to effectively target and eliminate pathogenic bacteria, limiting their therapeutic effectiveness. Additionally, the formation of aggregates may also increase the risk of inducing localized tissue damage or inflammation at the treatment site. Therefore, strategies to enhance the dispersibility and stability of CNT-based nanomaterials in biological fluids are essential to maximize their therapeutic potential and minimize potential adverse effects.

#### Other inorganic nanomaterials

Boron is an essential trace element known for its anti-inflammatory properties and its role in maintaining cell membrane stability [102,103]. Boron-derived compounds exhibit significant antibacterial effects and can even resist antibiotic resistance [104]. Table 4 provides a comparison of other inorganic photothermal nanomaterials and their antimicrobial activities.

**Table 4:** Comparison of the antibacterial activity of other inorganic photothermal nanomaterials

Nano-complexes	Size	Antibacterial mechanism	NIR laser	Bacteria	Effect	Ref.
B-QCS-BNN6	~200 nm	PTT/bacteria Capture/NO	808 nm 2.0 W/cm^2^	*E. coli* *S. aureus*	99% 99.9%	[105]
PU-GHB	60 μm	PTT/PDT	808 nm 1.5 W/cm^2^	*E. coli* *S. aureus* MRSA VER	>99.99%	[106]
Te-CTX NPs	50 nm	PTT	LED	MRSA	>90%	[107]
Nb_2_C NSs	100 ~ 200 nm	PTT	808 nm 1.5 W/cm^2^	*E. coli* *S. aureus*	90% 81%	[108]

**Figure 4:**
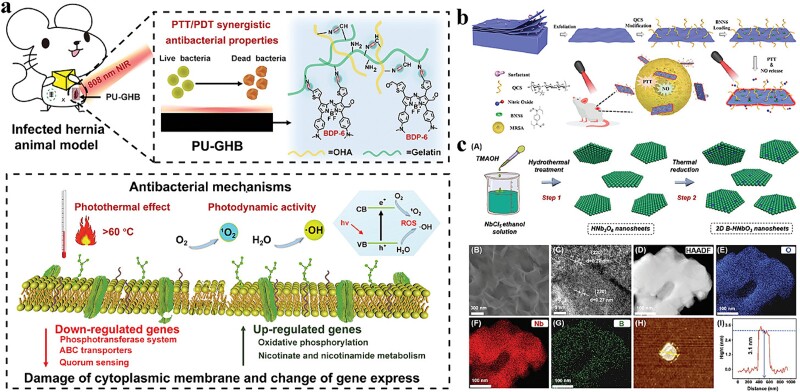
Antibacterial activity of other inorganic nanocomposites photothermal nanomaterials. (**a**) Antibacterial mechanism of PU-GHB. Adapted with permission [106]. Copyright © 1999–2023 John Wiley & Sons, Inc. All rights reserved. (**b**) Antibacterial mechanism of B-QCS-BNN6. Adapted with permission [105]. Copyright © Royal Society of Chemistry 2023. (**c**) Preparation and characterization of B-HNbO_3_ NSs. Adapted with permission [110]. Copyright © 1999–2023 John Wiley & Sons, Inc. All rights reserved. *PU* polyurethane

Lv *et al*. assembled 2D boron NSs (B NSs) with positively charged quaternary ammonium salts (QCS) and BNN6 to create a nanoplatform capable of trapping bacteria. The positive charge facilitated the capture of bacteria and enhanced the diffusion of NO to the bacterial surface (Fig. 4b). The B-QCS–BNN6 nanoplatform demonstrated the efficacy of PTT and the precise control of NO release under 808-nm laser stimulation. This combined treatment achieved >99.9% bacterial inactivation in just 5 min, thus enhancing the antibacterial function of PTT/NO [105]. Additionally, B-QCS–BNN6 nanoplatform was also used for wound treatment of MRSA infection *in vivo*. Wang *et al*. found that boron-doped TiO_2_ films driven by visible light could inhibit the growth of *E. coli*, *S. aureus*, and *Candida albicans* through the combined effect of photocatalysis and B doping [109]. Ren *et al*. found that B doping increased the specific surface area of NPs, promoted the separation of electrons and holes, and further improved the photocatalytic activity of nanomaterials (Fig. 4c). They used boron-doped niobate NSs (B-HNbO_3_ NSs) as an excellent photocatalytic antibacterial platform, generating a large amount of ROS under light conditions, thus significantly improving the antibacterial activity [110]. Zhang *et al*. developed a multimodal antimicrobial coating that combined PTT/PDT and BDP-6 based on a novel non-heavy-atom photosensitizer. A homogeneous coating was prepared on the polyurethane surface to obtain denoted as polyurethane (PU)-GHB by cross-linking BDP-6 (a kind of boron-dipyrromethene) with oxidized HA and natural polymer gelatin (Fig. 4a). PU-GHB exhibited excellent *in vitro* PTT/PDT synergistic antimicrobial performance against both sensitive and MDR bacteria [106]. This provided a promising strategy for the development of multifunctional antimicrobial coatings on implantable medical devices.

Studies indicated a crucial role of the size of NPs in their antibacterial activity. Generally, NPs with smaller particle sizes tend to exhibit higher antibacterial activity compared with those with larger particle sizes. The smaller size allows NPs to interact better with bacteria, enabling enhanced penetration into bacterial cells and increased surface area for effective antimicrobial action. This relationship between NP size and antibacterial activity highlights the importance of NP size optimization in developing effective antibacterial agents [111]. The bactericidal activity of tellurium NPs (Te NPs) against bacteria primarily resulted from the specific binding of Te oxygen anions to glutathione within bacterial cells. This binding triggered the reduction of TeO_3_^2−^ to Te (0), leading to its accumulation within the cells. Additionally, the Fenton reaction, facilitated by the presence of Te, generated ROS in the bacterial cells. The accumulation of ROS activated the intracellular oxidative stress response, ultimately leading to bacterial cell death. These mechanisms collectively contributed to the bactericidal effect of Te NPs against bacteria [112]. Indeed, the antibacterial performance of some currently synthesized Te NPs was not satisfactory in previous studies. However, given their excellent photothermal properties, these Te NPs needed to be combined with PTT for enhanced antibacterial applications. The combination of Te NPs with PTT led to improved antibacterial efficacy utilizing the photothermal effect of Te NPs, which involved converting absorbed light energy into localized heat. The combination of Te NPs and PTT offered a promising approach to overcome the limitations of Te NPs and enhance the overall antibacterial performance. Wu *et al*. prepared Te NPs loaded with cefotaxime (CTX) to restore the antibacterial activity of the ineffective β-lactam antibiotic CTX against MRSA. The *in vivo* evaluation showed that Te-CTX NPs combined with light accelerated the healing of MRSA-infected wounds with biosafety [107].

Chen *et al*. developed a thermosensitive Nb_2_C-based hydrogel (Nb_2_C@Gel) with antioxidant and antibacterial activities to promote diabetic wound healing. The Nb_2_C@Gel system consisted of Nb_2_C and bis-Poly(lactide-co-glycolide) (polyethylene glycol) (PLGA-PEG-PLGA) triblock copolymers. The fabricated Nb_2_C NSs showed good biocompatibility in *in vitro* cytotoxicity and hemocompatibility assays as well as in *in vivo* toxicity assays. Also, the Nb_2_C NSs showed good NIR photothermal antibacterial activity against *S. aureus* and *E. coli* [108].

Furthermore, an additional challenge inherent in using boron-based nanomaterials for antimicrobial applications is the difficulty in achieving precise control over their synthesis and functionalization. Boron-based nanoplatforms often require intricate fabrication techniques and precise chemical modifications to tailor their properties for specific antibacterial applications. However, the complexity of these synthesis and functionalization methods can lead to variability in the composition, structure, and performance of the final product. Inconsistencies in synthetic parameters or functionalization strategies may result in heterogeneous nanomaterials with unpredictable antibacterial efficacy and biocompatibility profiles. Therefore, achieving reproducible and scalable synthesis processes for boron-based nanomaterials is a significant challenge that needs to be addressed to facilitate their widespread application in antimicrobial therapies.

#### Polymer-based nanomaterials

Antimicrobial nanomaterials with photothermal effects were prepared using some conjugated polymers such as polyaniline (PANI), poly(3,4-ethylenedioxythiophene) (PEDOT), and PDA [113,114]. These nanopolymer materials have gained significant attention in the field of PTT due to their desirable properties, including biodegradability, high PCE, photostability, and simple synthesis processes. The biodegradability of nanopolymer materials is advantageous for biomedical applications because it allows for their safe use in the body without leaving long-lasting residues. Their high PCE signifies their ability to efficiently convert absorbed light energy into heat, resulting in effective PPT. This characteristic ensures maximum heat production for the targeted destruction of diseased cells while minimizing potential damage to healthy cells. Furthermore, nanopolymer materials are known for their photostability, stability, and efficient heat generation even under prolonged light exposure. This property ensures consistent therapeutic effects during PTT. In addition, the straightforward synthesis process of nanopolymer materials facilitates their scalable production and potential customization for specific applications. However, nanopolymers such as polyaniline gradually lose their NIR photothermal effect at physiological pH with time due to the shedding or neutralization of adsorbed protons [8]. Table 5 summarizes the comparison of polymer-based photothermal nanomaterials and their antibacterial activity.

**Table 5:** Comparison of antibacterial activity of polymer-based photothermal nanomaterials

Nano-complexes	Size	Antibacterial mechanism	NIR laser	Bacteria	Effect	Ref.
SF-CS-PDA	-	PTT/bio-chemo	808 nm 2.0 W/cm^2^	*S. aureus* *E. coli*	-	[115]
BSr@PPE	300 nm	PTT	808 nm 1.0 W/cm^2^	*S. aureus* *E. coli* MRSA	-	[116]
mPDA@DFO@CP-SNO	260 nm	PTT/NO	808 nm 1.0 W/cm^2^	*S. aureus* *E. coli*	100%	[117]
SF/PANI/CuS	-	PTT/PDT	808 nm 200 mW/cm^2^	*E. coli* *S. aureus*	99.9%	[118]
GG@PANI(Fe)-borax	173 nm	PTT/CDT/CT	1064 nm 0.5 W/cm^2^	*S. aureus* *E. coli*	100% 97.1%	[119]
PTNP	120 nm	PTT	808 nm 0.7 W/cm^2^	*S. aureus* *E. coli*	96.8% 97.5%	[120]
PSNO@IR780	100 nm	PTT/NO	808 nm 1.0 W/cm^2^	*S. aureus* *E. coli*	99.97% 99.99%	[121]
DPVA	-	PTT	UV	*S. aureus* *E. coli* MRSA EIEC	~99%	[122]
CP-F8P NPs	54 nm	PTT	808 nm 1.0 W/cm^2^	*E. coli* *S. aureus* MRSA *C. albicans*	99.3% >99% >99% >99%	[123]

PDA demonstrates a high PCE, enabling efficient conversion of absorbed light into heat for effective photothermal therapy. Its biocompatibility is crucial for its safe use in biomedical applications because it minimizes potential adverse reactions or toxicity concerns when interacting with biological systems. Furthermore, the hydrophilic nature of PDA facilitates its dispersibility in aqueous environments, making it suitable for various biomedical applications. Additionally, PDA exhibits excellent versatility in terms of modification, allowing for the introduction of functional groups or the incorporation of other therapeutic agents to enhance its therapeutic efficacy. These properties collectively position PDA as a promising candidate material for PTT, with the potential to overcome limitations and improve therapeutic outcomes in various biomedical applications [124]. Han *et al*. developed mussel-inspired cryogels based on chitosan and serine protein and functionalized them with NIR light-responsive PDA NPs. This versatile platform was used for regulating the wound microenvironment and promoting effective wound healing. Silk fibroin-chitosan-polydopamine (SF-CS-PDA) cryogels displayed significant photothermal antibacterial activity against both *E. coli* and *S. aureus* after 15 min of NIR. The magnitude of this activity depended on the concentration of PDA NPs incorporated into the cryogels [115]. Chen *et al*. developed a bioactive Si-Ca-Sr glass-based therapeutic regenerative nanohybrid (BSr@PPE), which demonstrated concentration-dependent photothermal effects, effective free-radical scavenging, antibacterial properties, UV-shielding capabilities, and high biocompatibility. It exhibited potent antibacterial activity against both normal and multidrug-resistant bacteria and encouraged fibroblast migration *in vitro*. *In vivo* animal experiments showed that BSr@PPE effectively promoted normal wound epithelial reconstruction, collagen deposition, and angiogenesis. Furthermore, it reduced inflammation and enhanced the repair process in multidrug-resistant bacteria-infected wounds. Additionally, it accelerated healing in tumor-damaged wounds by inhibiting tumor cells [116]. This study might provide new strategies and multifunctional bioactive compounds for treating multiple pathological tissue repair and regeneration. Liu *et al*. loaded deferoxamine (DFO) into mesoporous PDA (mPDA). Functionalized strong electrostatic interaction was achieved using third-generation polyamide amine polymers with the grafting terminal of chitosan S-nitroso-mercaptan (CP-SNO). Also, the multifunctional nanocomposite mPDA@DFO@CP-SNO was obtained, which exhibited mild photothermal therapy and controlled release of NO and DFO simultaneously under NIR laser irradiation. The synergistic mild photothermal therapy and NO antimicrobial effects of mPDA@DFO@CP-SNO effectively eliminated *E. coli* and *S. aureus*, as well as the biofilms formed by both bacteria [117].

**Figure 5:**
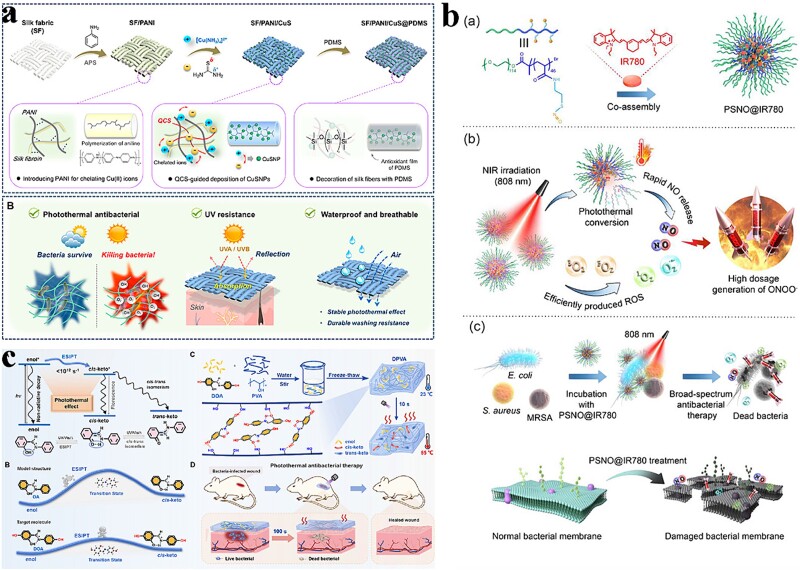
Antibacterial activity of polymer-based nanocomposites photothermal nanomaterials. (**a**) Schematic diagram of SF/PANI/CuS@PDMS nanofabric design and preparation process. Adapted with permission [118]. Copyright © 2023, American Chemical Society. (**b**) Preparation of PSNO@IR780 composite nanomaterials and schematic diagram of NO gas sterilization. Adapted with permission [121]. Copyright © 2023 Acta Materialia Inc. Published by Elsevier Ltd. All rights reserved. (**c**) Schematic diagram of the design and manufacture of DPVA hydrogel and its application. Adapted with permission [122]. Copyright © Royal Society of Chemistry 2023

Ren *et al*. employed PANI to modulate the deposition of CuS NPs on silk fabrics (SF), resulting in the construction of multifunctional textiles with efficient and stable photothermal properties. Fig. 5a illustrates this process. PANI, a conjugated polymer with photothermal properties, chelated Cu (II) ions and was polymerized by radicals, allowing the absorption of its molecules onto the fiber surface through intermolecular forces. Subsequently, the SF/PANI fabric bound with Cu ammonia ions through chelation and further interacted with chitosan quaternary ammonium salt-loaded thiourea, forming organic–inorganic hybrids known as PANI/CuSNPs. Finally, the fibers were encapsulated with polydimethylsiloxane (PDMS) to provide oxidation resistance and self-cleaning capability to the resulting photothermal textiles. This method enabled the construction of multifunctional textiles with efficient and stable photothermal properties; this was achieved by modulating the CuS NP deposition using PANI and subsequently encapsulating the NPs with PDMS [118]. The results showed that the SF/PANI/CuS composite textiles achieved satisfactory UV resistance. The inactivation rate of *S. aureus* and *E. coli* reached 99.9% under 200 mW/cm^2^ light, and a similar level remained after 10 times of washing. Huang *et al*. constructed GG@PANI(Fe)-borax hydrogels by linking Fe-doped polyaniline [PANI(Fe)] with guar gum (GG) chains. GG@PANI(Fe)-borax hydrogels with NIR-II-responsive photothermal conversion exhibited promising photothermal efficiency and controlled NIR-triggered drug release. The efficiency of the obtained hydrogels against *S. aureus* and *E. coli* was 97.1% [119]. Besides, these scaffolds incorporating GG@PANI(Fe) improved fibroblast proliferation and angiogenesis and accelerated wound repair in ruffled and infected wounded mice.

Additionally, other classes of polymer-based photothermal nanomaterials also exist. For example, Wu *et al*. designed a novel conjugated polymer called PDPP-TP, which featured a donor (D)–acceptor (A) structure with two alternating D–A pairs: thiophene (T)-diketopyrrolopyrrole (DPP) and thiophene (T)-thieno [3, 4-b] pyrazine (TP). The polymer was synthesized through direct aromatization polycondensation. The hydrophilic PDPP-TP-based NPs (PTNPs) were formed through self-assembly using DSPE-mPEG 2000 as a polymer matrix to enable efficient photothermal antimicrobial treatment. These NPs had a hydrated diameter of ~120 nm. PTNP exhibited strong absorbance in the NIR region and demonstrated a high PCE of 52.8% when subjected to 880-nm laser irradiation. In summary, Wu *et al*.’s design of PDPP-TP and the subsequent formation of PTNP exhibited excellent properties for efficient photothermal antimicrobial therapy, including strong NIR absorption and highly efficient photothermal conversion [120]. Jiang *et al*. designed PSNO@IR780 NPs (Fig. 5b), which could achieve effective bacterial inhibition based on the rapid release of NO induced by local temperature increase and the synergistic reaction of superoxide anion radicals generated by IR780 [121]. Yao *et al.* promoted the photothermal effect by introducing excited intramolecular proton transfer, with a temperature increase of nearly 30°C within 10 s of NIR irradiation (Fig. 5c). They showed excellent antimicrobial effects against drug-resistant bacteria, with <2 min of irradiation producing therapeutic effects on infected skin wounds [122]. Yuan *et al*. designed NIR-absorbing A–D–A-type conjugated oligomers with tunable backbones to modulate their photothermal conversion. The PCE of the conjugated oligomer CP-F8P NPs containing strong electron-donating components was the highest (81.6%). Phototherapeutic CP-F8P NPs effectively promoted wound healing in mice with diabetes and had good biocompatibility [123].

#### Organic small-molecule nanomaterials

In antibacterial PTT, metal-based, carbon-based, and polymer-based nanomaterials have demonstrated effective antimicrobial effects. However, their clinical translation is challenging due to the following factors. (1) High cost: the production of some nanomaterials used in PTT, particularly metal-based ones such as Au NPs, Ag NPs, or Cu NPs, can be expensive, making their widespread clinical adoption difficult. (2) Insufficient photothermal activity: certain nanomaterials may have limited PCE, which reduces their effectiveness in generating the necessary heat to achieve desired antibacterial outcomes. (3) Low biodegradability: the biodegradability of nanomaterials is a crucial factor for their safe use within the body. Some nanomaterials may have limited or slow degradation properties, leading to long-term accumulation and adverse effects. (4) High toxicity: certain nanomaterials, especially some metal-based ones, can exhibit high toxicities or induce immune responses in the body. This toxicity raises concerns about their safety in clinical applications.

Addressing these challenges and optimizing the properties of nanomaterials are essential considerations for the successful clinical translation of antibacterial PTT. Research efforts are focused on developing cost-effective, highly efficient, biodegradable, and biocompatible nanomaterials to improve the clinical applicability of antibacterial PTT [9]. Organic small-molecule dyes such as indocyanine green (ICG), Prussian blue (PB), black phosphorus (BP), and red phosphorus (RP) possess desirable characteristics of biocompatibility and biodegradability. These properties make them promising candidates for clinical applications in antimicrobial PTT. Of the aforementioned dyes, ICG and PB have already gained approval from the US Food and Drug Administration (FDA) for medical imaging purposes and the treatment of patients with internal contamination caused by exposure to cesium and/or thallium. The approval of ICG and PB by the FDA further supports the safety and efficacy of these dyes, paving the way for their potential use in the clinical applications of antimicrobial PTT. Research and development efforts continue to explore the potential of these organic dyes, as well as other small-molecule dyes, in the field of photothermal therapy for combating various microbial infections. Table 6 summarizes the organic small-molecule nanomaterials and their comparative antibacterial activities.

**Table 6:** Comparison of the antibacterial activity of organic small-molecule nanomaterials with photothermal effect

Nano-complexes	Size	Antibacterial mechanism	NIR laser	Bacteria	Effect	Ref.
GFNPs	171 ~ 187 nm	PTT/PDT	808 nm, 2.0 W/cm^2^	*P. aeruginosa*	~100%	[125]
AC-PB	94.8 nm	PTT	980 nm 2.0 W/cm^2^	*S. aureus* *E. coli*	76% 75%	[126]
BP/Gel	100 ~ 200 nm	PTT	808 nm, 1.0 W/cm^2^	*S. aureus*	98%	[127]
PCL/AgNPs/BP	-	PTT/Ag^+^	808 nm, 0.5 W/cm^2^	MRSA *E. coli*	95.51 ± 1.67% 99.23 ± 0.06%	[128]
BP@CQDs	120 nm	PTT/PDT	808 nm, 1.5 W/cm^2^	*E. coli* *S. aureus*	91.7% 98.4%	[129]
Phthalocyanines	-	PTT/PDT	680 nm 0.5 W/cm^2^	*C. albicans* *E. coli*	-	[130]

**Figure 6:**
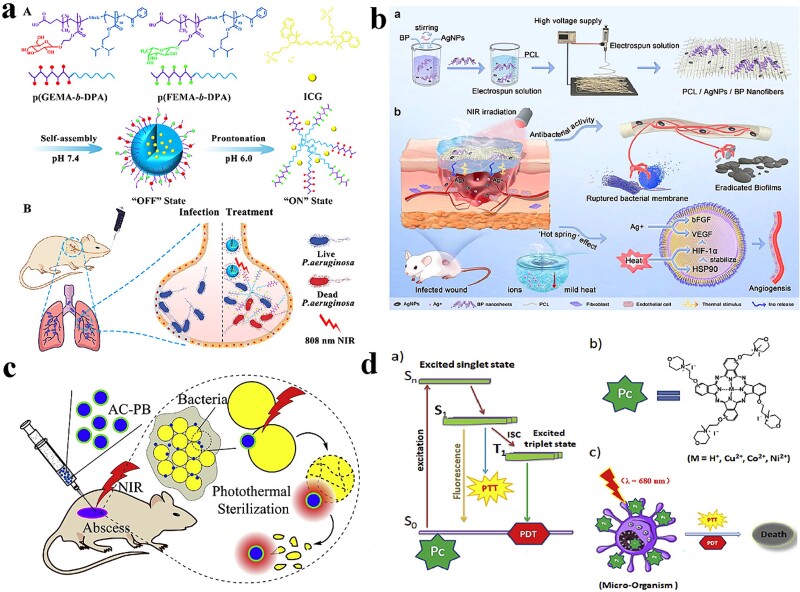
Antibacterial activity of organic small-molecule photothermal nanomaterials. (**a**) Design and preparation of GFNPs and applications. Adapted with permission [125]. Copyright © 2019, American Chemical Society. (**b**) Schematic illustration of the preparation and application of PCL/AgNPs/BP nanofibers. Adapted with permission [128]. Copyright © 2023 the author(s). Published by Elsevier Ltd. (**c**) Antibacterial mechanism of AC-PB. Adapted with permission [126]. Copyright © 2023 Elsevier Ltd. All rights reserved. (**d**) Antibacterial mechanism of phthalocyanines. Adapted with permission [130]. Copyright © 2023 Elsevier Ltd. All rights reserved. *AC-PB* acetylcysteine-modified PB

In a recent clinical trial, ICG was used for PTT in treating periodontitis. ICG exhibited advantages over chlorhexidine gel in terms of bacterial resistance and cytotoxicity to oral cells. ICG demonstrated reduced bacterial resistance compared with chlorhexidine gel, implying that bacteria were less likely to develop resistance to ICG treatment. This was an essential factor in the long-term effectiveness of antimicrobial therapies. Furthermore, ICG showed lower cytotoxicity toward oral cells compared with chlorhexidine gel. This suggested that ICG had a higher degree of biocompatibility and was less harmful to the surrounding healthy oral tissue during treatment [131]. Zhao *et al*. used a pH-sensitive polymer with a hydrophobic core to encapsulate ICG (Fig. 6a). This polymer system demonstrated pH-responsive behavior, where the core transformed into a hydrophilic state when exposed to acidic conditions (pH = 6). The protonation of the polymer core triggered the conversion into a hydrophilic block under acidic conditions, resulting in the controlled release of ICG. The release of ICG reached up to 70% within 36 h. In contrast, the release of ICG was limited to only 16% within 48 h under neutral conditions (pH 7.4). These findings indicated that this pH-responsive system could selectively release the PTA, in this case, ICG, at acidic infection sites. This targeted release mechanism allowed for specific PTT at the infection site, minimizing potential damage to surrounding normal tissues. Using the pH-sensitive polymer and its controlled-release properties, Zhao *et al*.’s study contributed to the development of systems that could enhance the efficacy and safety of specific PTT by minimizing off-target effects on healthy tissues [125]. Cai *et al*. used acetylcysteine-modified PB NPs (AC-PB) for effective photothermal sterilization in a concentration-dependent manner under a 980-nm NIR laser (Fig. 6c). The inhibition rates of AC-PB (50 μg/ml) against *S. aureus* and *E. coli* were 74 and 75%, respectively. The antibacterial mechanism suggested that AC disrupted bacterial basal biofilms, whereas the NIR-driven photothermal effect disrupted lipids on cell membranes [126].

BP and RP have garnered significant attention in PTT because of their remarkable photothermal effects and nontoxic degradation products under physiological conditions. BP, a 2D material, possesses a high PCE, making it an effective photothermal agent for PTT applications. Its unique layered structure promotes strong light absorption and efficient conversion of light energy into heat, enabling the targeted destruction of pathogens. Similarly, RP has also demonstrated excellent photothermal properties. It can efficiently convert NIR light into heat, allowing for precise thermal ablation of targeted tissues or microorganisms. Importantly, both BP and RP produce nontoxic degradation products under physiological conditions. These materials break down into nontoxic phosphorus derivatives that can be naturally eliminated by the body without any adverse effects. This characteristic enhances their potential for safe and biocompatible PTT in clinical applications [132–135]. Indeed, BP-mediated NIR photothermal therapy has emerged as a promising approach for clinical applications in treating bacterial infections. BP demonstrates high PCE, allowing for the effective conversion of NIR light into heat for targeted bacterial destruction. Moreover, BP-based photothermal therapy lacks bacterial resistance, which is crucial for long-term efficacy. Miao *et al*.’s work involved fabricating a hydrogel scaffold incorporating BP NSs, resulting in potent antibacterial properties. The BP-based hydrogel achieved a remarkable killing rate of >98% against *S. aureus* under NIR radiation. This highlighted the potential of BP-based hydrogels as effective antibacterial agents in PTT. The biodegradability of small molecules such as BP is a significant advantage for practical applications in antibacterial PTTs. The ability of these molecules to be naturally degraded and eliminated by the body mitigates concerns about their long-term accumulation and potential toxicity [127]. Zhao *et al*. developed a bifunctional wound dressing with strong antimicrobial properties. They prepared PCL/AgNPs/BP nanofibers through electrospinning, with polycaprolactone (PCL) serving as the carrier material (Fig. 6b). When applied to a full-skin injury model, this wound dressing could eliminate bacterial biofilms, stimulate cell proliferation, and promote angiogenesis [128]. Liu *et al*. established an antimicrobial platform with synergistic PTT and PDT, which exhibited excellent antibacterial properties against *S. aureus* and *E. coli*. Additionally, BP@CQDs exhibited good hemocompatibility, cytocompatibility, and biocompatibility during *in vivo* treatment [129]. This study demonstrated the potential of BP NSs for a wide range of applications in infectious microenvironments and developed a potential strategy for wound repair in *S. aureus* infections.

Another class of organic small-molecule compounds, such as phthalocyanines, has also been extensively studied. Fan *et al*. synthesized a series of *a*-tetrasubstituted phthalocyanines (M = H^+^, Cu^2+^, Co^2+^, Ni^2+^) with a morpholine group and their corresponding quaternized derivatives (Fig. 6d). The spectroscopic and photosensitive properties, photothermal effects, aggregation trends, and partition coefficients of these phthalocyanines were evaluated. The killing effect of cationic phthalocyanines on *E. coli* and *C. albicans* was better than that of neutral analogues under NIR light irradiation (680 nm) [130].

### Materials relying on drug delivery for antibacterial action

#### Polymer-based nanomaterials

Polymer-based NPs play a key role in drug delivery, offering advantages for various applications, including drug encapsulation and release, targeted delivery, and controlled release and potentiation. In controlled-release systems, polymer NPs are commonly used to load antimicrobial drugs or biologically active substances into them and achieve slow and targeted release of drugs by modulating the structure and chemical composition of the particles [136]. When the polymer NPs come into contact with the wound tissue, the drug is gradually released from the carrier and penetrates around the wound at a certain rate, interacting with the bacteria and exerting an antimicrobial effect [137,138]. Additionally, polymer-based NPs have the ability of targeted delivery, achieved through surface modification or functionalization, for selective recognition and localization to specific cells or tissues. This targeted delivery can increase the local concentration of the drug and reduce damage to healthy tissues, thereby improving therapeutic efficacy and reducing side effects. Feng *et al*. designed an antibiotic nanocarrier from quaternary ammonium-based hyperbranched polyureas to transport the antibiotic rifampicin inside the biofilm, which exhibited synergistic antimicrobial effects in killing planktonic bacteria and eradicating the corresponding biofilm and showed excellent efficacy in promoting wound healing [139].

Polymer-based NPs can also be used to release drugs slowly under specific conditions through controlled release and potentiation, thus prolonging the duration of drug action, reducing the frequency of medication, and improving patient compliance and therapeutic efficacy. At the same time, polymer-based NPs can also enhance the bioavailability and cellular uptake of drugs through the nanoscale effect, further improving the therapeutic efficacy of drugs [140]. Yang *et al*. designed a multifunctional telomere (TD) platform that could efficiently load charged antibiotic molecules through a combination of multivalent and synergistic charge and hydrophobic interactions. The coupling of TDs in biocompatible hydrogels allowed for localized application to provide sustained antibiotic release [141]. Puleo *et al*. produced polymer particles by electrospraying with polybutyl succinate (PBS) and incorporating water-insoluble ciprofloxacin into the polymer matrix. PBS is a well-known water-insoluble polymer with tunable chemical and physical properties and is also used for tissue regeneration and wound healing applications. It can inhibit bacterial replication and effectively treat various infections [142]. The use of polymer-based NPs in drug delivery offers many advantages, but also presents challenges in terms of stability and storage. These particles are susceptible to external environmental factors leading to drug inactivation or destruction of the particle structure, thus reducing drug effectiveness.

#### Liposomes

Another commonly used drug delivery vehicle is a nanocapsule carrier consisting of a monolayer or multilayer lipid membrane, mainly composed of hydrophilic and hydrophobic layers [143]. Antimicrobial drugs can be encapsulated inside these carriers, and the slow release of drugs can be achieved through the structure of the lipid layer. Compared with polymer NPs, liposomes have a higher drug-loading capacity and more flexible release characteristics. They can fuse with the plasma membrane of microbial cells and release high concentrations of drugs into the cell membrane or cytoplasm, achieving more efficient delivery and avoiding the increase of drug efflux [144,145]. Additionally, liposomes can be easily coupled to antibodies, proteins, or enzymes, enabling the administration of specific substances for targeted delivery, making them potential candidates for targeted antibiotic delivery [146].

Feng *et al*. formed rColMA/QCSG/LIP@AS/Ag@MOF (RQLAg) hydrogels by modifying recombinant collagen and quaternary ammonium chitosan, and incorporating Ag NPs with antimicrobial properties (Ag@MOF) and *Centella asiatica* glycoside liposomes (Lip@AS). *Centella asiatica* encapsulated by liposomes was released in the wound environment to promote angiogenesis and played an essential role in promoting the expression of mRNAs for angiogenic markers [147]. Although liposomes are commonly used as carriers for antibiotic drugs, conventional liposomes do not have a strong affinity for biofilms when it comes to treating biofilms. Hence, the development of nano-formulations such as liposomes with the ability to effectively traverse biological barriers becomes crucial. Xie *et al*. used liposome encapsulation with a high cholesterol concentration, which could effectively remove bacterial biofilms by adjusting the drug loading [148].

## Conclusions

Nanomaterials represent a diverse and promising arsenal in combating bacterial infections. Among them, polymer-based nanomaterials stand out due to their versatility and tunability, offering innovative solutions for antimicrobial applications. These materials can be precisely engineered for controlled release, targeted delivery, and sustained efficacy, which address the challenges faced by metallic NPs such as cytotoxicity and stability. Polymer-based nanomaterials can encapsulate antimicrobial agents, protecting them from degradation and ensuring controlled release, thus enhancing therapeutic efficacy and improving patient compliance. Additionally, surface modification allows for targeted delivery, increasing the local concentration of antimicrobial agents at infection sites while minimizing exposure to healthy tissues and reducing potential side effects. Moreover, these nanomaterials can respond to specific stimuli, enabling on-demand drug release triggered by environmental cues like pH, temperature, or enzymatic activity, thereby enhancing the precision and efficacy of antimicrobial treatments. The tunability, biocompatibility, controlled-release capabilities, and targeted delivery options of polymer-based nanomaterials highlight their potential as a platform for developing next-generation antimicrobial therapies. Continued research and innovation in polymer chemistry and nanotechnology are essential to further enhance the effectiveness and safety of these materials, ultimately leading to improved outcomes in the fight against bacterial infections.
